# Household transmission investigation for Corona Virus Disease 2019 (COVID-19) in a rural and urban population of north India

**DOI:** 10.1371/journal.pone.0287048

**Published:** 2023-10-05

**Authors:** Kapil Yadav, Subhashini K. J., Suneeta Meena, Rakesh Kumar, Ravneet Kaur, Mohan Bairwa, Shashi Kant, Puneet Misra, Sanjay K. Rai, Mohammad Ahmad, Anisur Rahman

**Affiliations:** 1 Centre for Community Medicine, All India Institute of Medical Sciences, New Delhi, India; 2 Consultant, WHO Unity Project, Centre for Community Medicine, All India Institute of Medical Sciences, New Delhi, India; 3 Laboratory Medicine, Microbiology Division, All India Institute of Medical Sciences, New Delhi, India; 4 National Professional Officer (Research), World Health Organization Country Office for India, New Delhi, India; 5 Health Emergencies and Research Officer, World Health Organization Country Office for India, New Delhi, India; Centers for Disease Control and Prevention, UNITED STATES

## Abstract

**Background:**

Transmissibility within closed settings, such as households, can provide a strategic way to characterize the virus transmission patterns because the denominator can be well defined. We aimed to characterize the household transmission of Severe Acute Respiratory Syndrome Coronavirus (SARS CoV-2) and its associated risk factors.

**Methods:**

This prospective case-ascertained study was conducted among the household contacts of laboratory-confirmed SARS CoV-2 cases residing in Ballabgarh, Haryana. We enrolled 148 index cases and their 645 household contacts between December 16, 2020 and June 24, 2021. We defined household contact as any person who had resided in the same household as a confirmed COVID-19 case. Baseline data collection and sample collection for real time- reverse transcriptase polymerase chain reaction (RT-PCR) and IgM/IgG against SARS CoV-2 were done on day 1 visit, and followed for a period of 28 days. RT-PCR was repeated on day 14 or whenever the contact is symptomatic and blood sample for serology was repeated on day 28. We estimated household secondary infection rate (SIR) and other epidemiological indicators–median incubation period and serial interval. We employed binomial logistic regression to quantify risk factors associated with infection.

**Results:**

The household SIR was 30.5% (95% CI: 27.1–34.1%). The secondary clinical attack rate was 9.3% (95% CI: 7.2–11.8). The risk factors that showed higher susceptibility to infection were household contacts who were the primary care giver of the case, whose index cases were symptomatic, those with underlying medical conditions, those living in overcrowded households, who were sharing toilet with the index cases and also who were not wearing a mask when coming in contact with the case. The median (IQR) incubation period was 4 days (4, 5), mean (SD) serial interval 6.4 (±2.2) days, and median (IQR) serial interval 5 days (5, 7).

**Conclusion:**

Households favour secondary transmission of SARS CoV- 2, hence, index cases are recommended to self-isolate and wear masks; and household contacts to follow strict COVID infection control measures within households when a family member is infected.

## Introduction

The Coronavirus Disease 2019 (COVID-19) caused by Severe Acute Respiratory Syndrome Coronavirus 2 (SARS-CoV-2) spreads via direct and/or indirect contact with the infected people [1]. With an alarming increase in the number of cases reported globally, World Health Organization (WHO) declared COVID 19 as a pandemic on 11^th^ March, 2021 [2]. The number of COVID cases continued to escalate, reporting more than 750 million cases globally (7^th^ March, 2023). India stood second in the highest number of cases reported after USA [3].

SARS CoV- 2 is a novel virus with continuously evolving transmission trends. The detection and spread of such novel virus are accompanied by uncertainty over the key epidemiological, clinical and virological characteristics and dynamics of the disease transmission [4]. Crowded indoor environments with sustained close contacts such as households, are a particularly high-risk setting for disease transmission [1]. The advantage of choosing households is that they have a defined population that may not mix readily with the larger surrounding community. Therefore such settings can provide a strategic way to characterize virus transmission patterns because the denominator is often well defined. Also, exposure is within the setting, and follow-up of household contacts is generally more feasible in this well-defined setting than in an undefined one [4]. In low and middle-income countries (LMICs), where a quality healthcare service for the entire population has been a challenge, overcrowding and inadequate housing per person can enhance the risk of transmission among close contacts in households [5].

The disease transmissibility can be characterized by household Secondary Attack Rate (SAR) [1]. In epidemiology, a household secondary attack rate is defined as number of household cases occurring within the incubation period upon exposure to a primary case divided by total susceptible household contacts [6]. This indicator will help in studying and understanding the transmission dynamics of infectious diseases and also provide information regarding how social interactions influence transmission risk [7].

To date, the transmissibility of the disease has primarily been assessed at the population level, using mathematical models, or at the individual level in synthetic populations using agent-based models coupled with statistical methods to capture the current evidence [8]. Literature shows that secondary transmission or infection of SARS-CoV-2 in household contacts ranges from 4.6% to 49.6% [1, 6, 9–11]. In India, transmissibility within households or close contacts remains under investigated. Only few studies have been conducted so far. A study by Laxminarayan et al. [12] in two south Indian states viz. Tamilnadu and Andhra Pradesh, reported that the per-contact risk of infection was 9.0% (7.5–10.5%) in the household. However, it was based on the assumptions of robust contact tracing and not entirely a household transmission study.

In accordance with the WHO Unity protocol 2020 on household transmission of COVID 19 [4], this study was undertaken with the objectives of estimating the secondary infection rate and secondary clinical attack rate for household contacts and factors associated with any variation in the secondary infection risk and to provide suggestions for possible public health measures.

## Methods

We conducted a prospective case- ascertained study of all identified household contacts of a RT- PCR confirmed SARS CoV-2 cases in the intensive field practice area of the Comprehensive Rural Health Services Project (CRHSP), AIIMS in Ballabgarh block of district Faridabad, Haryana. Faridabad district stood second with the highest number of COVID cases in Haryana with a case positivity rate of 5.8% at the start of the study and it further increased to 12.24% during the second wave of COVID-19 (April 2021). The intensive field practice area caters to around 1.0 lakh population in rural area and around 2.5 lakhs population in urban area of Ballabgarh.

We included the households of RT-PCR confirmed COVID-19 cases and their contacts irrespective of age and gender and who consented for the study, and excluded those households in which the date of symptom onset of COVID-19 was same for more than one family member (co-primary cases), all household members who tested positive on day 1 visit and who were serologically positive on day 1, hospitalized cases and their contacts, and children less than one-year old and those households who did not consent for the study. The first household was enrolled on 16^th^ December 2020, the last one was on 28^th^ May 2021 and the final follow-up date was 24^th^ June 2021. The Delta variant (or B.1.617.2 strain) of the coronavirus was the dominant strain at the time of the study.

### Sample size

The secondary attack rate as reported by Laxminarayan et al. [12] using contact tracing data was around 9–10%. Since household SAR would likely be higher, we took it like 15% with 20% relative precision. With a 10% non- response rate, the sample size was calculated as 623. Assuming the average household size in Ballabgarh, Faridabad as 5, a minimum of 125 households with a laboratory confirmed SARS CoV-2 index case were required (i.e., 125 laboratory confirmed cases and their 500 household contacts). In this study, 179 households with 757 household contacts were interviewed. Considering the exclusion criteria, 31 households and their 112 contacts were excluded. Thus 148 households and their 645 contacts were finally enrolled in this study.

This study was conducted in accordance with the WHO Unity Protocol 2020- Household transmission investigation protocol for coronavirus disease 2019 (COVID-19) [4].

### Operational definitions [4]

**Confirmed case of COVID 19:** A person with laboratory confirmation of SARS CoV-2 infection. **Household**: a group of people (two or more) living in the same residence. **Household contact**: any person who has resided in the same household as a confirmed COVID-19 case. **Secondary clinical attack rate**: A measure of the frequency of new symptomatic cases of COVID-19 infection that occur among contacts within the incubation period (14 days) following exposure to a primary confirmed case, in relation to the total number of exposed susceptible (who lacks resistance to a disease and at risk of becoming infected by a disease) contacts. **Secondary infection rate (SIR)**: A measure of the frequency of new infections of COVID-19 among contacts within the incubation period (14 days) following exposure to a primary confirmed case, in relation to the total number of exposed susceptible contacts. (The rate of contacts being infected, assessed through serological assays/RT PCR). **Incubation period**: The period between an exposure resulting in SARS CoV-2 infection and the appearance of the first sign or symptom of the disease. **Serial Interval**: The period from the onset of symptoms in the primary case to the onset of symptoms in a contact. **Severity of the COVID**: Mild—Cases with uncomplicated upper respiratory tract infection with fever, cough, sore throat, nasal congestion, malaise, headache without evidence of breathlessness or Hypoxia (normal saturation). Moderate—Pneumonia with no signs of severe disease with the presence of clinical features of dyspnoea and or hypoxia, fever, cough, including SpO2 <94% (range 90–94%) on room air, Respiratory Rate more or equal to 24 per minute. Severe—Severe pneumonia plus one of the following; respiratory rate >30 breaths/min, severe respiratory distress, SpO2 <90% on room air [13]. **Overcrowding**: It is assessed using the persons per room criterion. The number of persons in the household is divided by the number of rooms in the house. One room- 2 persons; two rooms—three persons; three rooms– 5 persons; four rooms–seven persons; five rooms–ten persons; more than five rooms–add two persons for each additional room [14].

### Data collection method

The list of RT–PCR confirmed index COVID cases were obtained from the Haryana Government’s COVID 19 portal and also through the Senior Medical Officers of the field practice area of Ballabgarh. The health care workers informed the index cases about their disease status, and all of their household contacts were quarantined in their houses immediately for 14 days. The included index cases were communicated telephonically to confirm their availability for a face-to-face interview. The enrolled index cases and their household contacts were paid four home visits, including the enrolment visit (Day 1) and three follow-up visits (Day 7, 14 and Day 28) within 28 days of enrolment.

### Data collecting tools

#### Questionnaire

Consenting individuals answered the questionnaire adapted from the WHO household transmission investigation protocol [4]. It covered demographic information, relationship with the known index case, exposure attributes during contact with the case or cases during the defined time interval and previous medical history. The date of onset and type of clinical symptoms experienced by the subject (even if presented individually), were also recorded. The questionnaire assessed the symptoms specifically suggestive of COVID 19, and other symptoms also. Data collection was done using Epicollect 5 mobile and web based application.

#### Sample collection

All baseline upper respiratory tract specimens (nasopharyngeal/ oropharyngeal swab) for RT-PCR and blood samples for serology were collected at the initial home visit (Day 1) from all household contacts, regardless of symptoms. The contacts were followed up and nasopharyngeal/oropharyngeal swab was collected whenever the contacts showed any symptoms of COVID 19. If otherwise, a nasopharyngeal/oropharyngeal swab was collected on day 14 from all household contacts, for virological testing, regardless of symptoms. And at the day 28 visit, a blood sample was collected from all household contacts for Enzyme Linked Immunosorbent Assay (ELISA).

RT-PCR, the gold standard technique was employed for virological diagnosis of SARS CoV-2. And ELISA was performed using the WANTAI Kit (Beijing Wantai Biological Pharmacy Enterprise Co., Ltd, China) for serological testing. WANTAI SARS-CoV-2 Antibody ELISA is for the qualitative detection of total antibody (including IgM and IgG) to SARS-CoV-2 in serum samples with a sensitivity of 94.4% and specificity of 100% [15].

All those involved in collecting and transporting specimens were trained in safe handling practices and spill decontamination procedures. For each biological sample collected, the time of collection, the conditions for transportation, and the time of arrival at the study laboratory were recorded. Nasopharyngeal/oropharyngeal specimens were transported to the Laboratory Medicine, AIIMS, New Delhi within 48 hours of collection, maintaining the cold chain. Serum was separated from whole blood and stored in Ballabgarh laboratory and shipped at 2–8°C. The results of the tests were communicated to the study participants.

### Ethical considerations

AIIMS Ethics Committee clearance was obtained and the guidelines about the participant information sheet (PIS) and Participant informed consent form (PICF) were followed. Permission from the Medical officers of the respective areas was obtained before the start of the study. The purpose of the investigation was explained to all the household contacts of the index cases in the local language (Hindi). Written informed consent was obtained from each study participant willing to participate in the study. For 1–11 years- parents’ consent was taken, for 12–18 years parent’s consent and participant’s assent, and for more than 18 years participant’s consent was taken.

### Statistical analysis

Epicollect 5 and Microsoft Excel were used for data management. Data analyses were conducted using Stata 16 (Stata Corp, Texas). Descriptive statistics were employed. Continuous variables were expressed as mean (standard deviation) and median (interquartile ranges), and categorical variables as percentages (%). SIR was calculated by dividing number of secondary cases by the total exposed susceptible contacts; Secondary clinical attack rate by dividing the number of only symptomatic secondary cases by the total exposed susceptible contacts. We calculated individual Secondary Infection Rate for univariate analyses of case and household factors, to examine transmission risk. To explore the risk factors associated with COVID-19 among household contacts, a binomial regression model and post-estimation method were used.

## Results

We contacted 179 households with 757 household contacts during the study period. Out of which, 31 households with index case and their 112 contacts were excluded, applying the exclusion criteria. The remaining 148 households and their 645 contacts were eligible to be included in the study. The follow up rate for the three subsequent visits were 93.8%, 100%, 91.3% and 83.6% respectively.

The demographic and baseline characteristics of the participants are described in Table 1. Of the 645 household contacts, 197 contacts (30.5%) became secondary cases. The median (IQR) age of the index cases was 39 years (28, 53) and that of the secondary cases was 30 years (17,52). The majority of the index cases (67.6%) and secondary cases (52.3%) were in the age group of 20–59 years. Among the index cases, 56.1% were symptomatic. Majority (69.5%) of the secondary cases were asymptomatic.

**Table 1:** Demographic and baseline characteristics of index cases, household contacts, and secondary cases of COVID in CRHSP, Ballabgarh, Haryana, 2021.

Demographic Characteristics		Index cases (n = 148)	Household contacts (n = 645)	Secondary cases (n = 197)
**Age**	Median (IQR)	39 (28,53)	30 (17,48)	30 (17,52)
		n (%)	n (%)	n (%)
**Age Group (in years)**	<10 years	0 (0.0)	63 (9.8)	20 (10.2)
	10–19 years	8 (5.4)	127 (19.7)	36 (18.3)
	20–59 years	112 (75.7)	379 (58.8)	109 (55.3)
	> 60 years	28 (18.9)	76 (11.8)	32 (16.2)
**Sex**				
	Male	97 (65.5)	300 (46.5)	89 (45.2)
	Female	51 (34.5)	345 (53.5)	108 (54.8)
**Geographical Distribution**				
	Urban	106 (71.6)	397 (61.6)	114 (57.9)
	Rural	42 (28.4)	248 (38.4)	83 (42.1)
**Occupation**				
	Homemaker	35 (23.6)	195 (30.2)	59 (29.9)
	Government employee	8 (5.4)	9 (1.4)	5 (2.5)
	Private employee	69 (46.6)	55 (8.5)	13 (6.6)
	Student	20 (13.5)	226 (35.0)	70 (35.5)
	Health care worker	0 (0)	8 (1.2)	2 (1.0)
	Others	16 (10.9)	120 (18.6)	40 (24.8)
**Household size**				
	Household size (Median)	5 (3,6)	Not applicable	Not applicable
	< = 2	13 (8.8)		
	3–5	79 (53.4)		
	6–8	36 (24.3)		
	>8	20 (13.5)		
**Severity of the disease**				
	Asymptomatic	65 (43.9)	Not applicable	137 (69.5)
	Mild	69 (46.6)		56 (28.4)
	Moderate	13 (8.8)		4 (2.0)
	Severe	1 (0.7)		0 (0)
**Clinical symptoms** ^**#**^				
	Fever, Headache and Respiratory symptoms (Cough, Dyspnoea, Sore throat, Runny nose)	85 (57.4)	60 (9.3)	56 (28.4)
	Loss of smell and taste	23 (15.5)	0 (0)	35 (17.8)
	Aches and Fatigability	21 (14.2)	12 (1.9)	36 (18.3)
	Gastrointestinal symptoms (diarrhoea/vomiting)	2 (1.4)	1 (0.2)	17 (8.6)
**Underlying Medical Conditions** ^**#**^				
	None	109 (73.6)	569 (88.2)	165 (83.8)
	Diabetes	23 (15.5)	28 (4.3)	11 (5.6)
	Hypertension	17 (11.5)	39 (6.00	14 (7.1)
	Heart Disease	4 (2.7)	3 (0.4)	1 (0.5)
	Lung disease	3 (2.0)	5 (0.7)	4 (2.0)
	Others	2 (1.4)	21 (3.3)	9 (4.6)
**Sought medical care**				
	Yes	62 (41.9)	0 (0)	60 (30.5)
	No	86 (58.1)	645 (100.0)	137 (69.5)
**Hospitalization during follow up**				
	Yes	4 (2.7)	1 ((0.2)	4 (2.0)
	No	144 (97.3)	644 (99.8)	193 (98.0)
**Living Status during follow up**				
	Alive	145 (98.0)	645 (100.0)	197 (100.0)
	Dead	3 (2.0)	0 (0)	0 (0)

Figures in the parentheses indicate column %

# includes multiple answers from one respondent

The 148 index cases gave rise to 197 secondary cases among 645 household contacts. Hence the secondary infection rate was 30.5% (95% CI: 27.1–34.1). Among 197 secondary cases, 82 (41.6%) were identified through RT PCR and 115 (58.4%) were identified by serology on day 28 (ELISA positive and RT PCR negative). Also, secondary clinical attack rate, serial interval and incubation period were estimated from 60 symptomatic secondary cases. The secondary clinical attack rate was 9.3% (95% CI: 7.2–11.8). We estimated the median (IQR) incubation period of SARS CoV-2 was 4 days (4,5). The mean (SD) serial interval was 6.4 (±2.2) days and the median (IQR) serial interval was 5 days (5,7). The hospitalization rate among index cases was 2.7% (95% CI: 0.7–5.4) and that of secondary cases was 2.0% (95% CI: 0–4.0). The infection fatality rate among index cases was 2.0% (95% CI: 0.0–4.7). There were no fatalities among secondary cases.

The factors significantly associated with SIR in the univariate analysis included household contacts, those who were the primary care giver of the case, those who were with underlying medical conditions, overcrowded households, those whose index cases were symptomatic, those who were sharing toilet with the index cases, those who had physical contact with index cases and those who were not wearing a mask when coming in contact with the case (Table 2).

**Table 2:** Secondary infection rate of COVID-19 and its association with selected variables in households of CRHSP, Ballabgarh, Haryana, 2021.

Variables	No. of household contacts (n = 645)	No. of secondary cases (n = 197)	Secondary Infection Rate % (95% CI)	Unadjusted OR (95% CI)^#^	p value
**Age Group (in years)**					
<10 years	63	20	31.7 (20.3–43.2)	Ref	
10–19 years	127	36	28.3 (20.5–36.2)	0.85 (0.44–1.63)	0.629
20–59 years	379	109	28.8 (24.2–33.3)	0.86 (0.49–1.54)	0.629
> 60 years	76	32	42.1 (31.0–53.2)	1.56 (0.78–3.14)	0.210
**Sex**					
Male	300	89	29.7 (24.5–34.8)	Ref	
Female	345	108	31.3 (26.4–36.2)	1.08 (0.77–1.51)	0.652
**Geographical Distribution**					
Urban	397	114	28.7 (24.3–33.2)	Ref	
Rural	248	83	33.5 (27.6–39.3)	1.25 (0.85–1.75)	0.203
**Relationship to index case**					
Others	376	98	26.1 (21.6–30.5)	Ref	
Primary care giver	269	99	36.8 (31.0–42.5)	1.61 (1.43–2.84)	0.036*
**Duration of contact with the index case (in days)**					
<3 days	579	173	30.6 (26.7–34.6)	Ref	
>3 days	66	24	36.4 (24.6–47.9)	1.88 (0.86–4.09)	0.114
**Household size**					
<4	162	45	27.8 (20.9–34.7)	Ref	
4–6	198	72	36.4 (29.7–43.1)	1.46 (0.95–2.32)	0.084
>6	285	80	28.1 (22.6–32.9)	1.01 (0.66–1.55)	0.947
**Overcrowding**					
Absent	533	158	29.6 (25.8–33.5)	Ref	
Present	112	39	34.8 (26.0–43.6)	1.27 (1.02–1.95)	0.028*
**Clinical Severity of index case**					
Asymptomatic	264	75	28.4 (23.0–33.8)	Ref	
Mild	291	106	36.4 (30.9–42.0)	1.44 (1.01–2.06)	0.045*
Moderate/Severe	90	16	17.8 (09.8–25.7)	1.54 (1.29–2.99)	0.048*
**Underlying medical conditions**					
Absent	569	165	28.9 (25.3–32.7)	Ref	
Present	76	32	42.1 (31.0–53.2)	1.78 (1.09–2.91)	0.021*
**Sharing a room**					
Absent	624	191	30.6 (27.0–34.2)	Ref	
Present	21	6	28.6 (9.2–47.9)	0.91 (0.35–2.37)	0.842
**Sharing a meal**					
Absent	638	196	30.7 (27.1–34.3)	Ref	
Present	7	1	14.2 (11.6–40.2)	0.36 (0.04–3.14)	0.366
**Sharing utensils**					
Absent	639	195	28.3 (23.9–32.7)	Ref	
Present	6	2	37.4 (2.6–77.4)	1.13 (0.21–6.27)	0.882
**Sharing a toilet**					
Absent	139	35	28.9 (10.9–46.8)	Ref	
Present	506	162	36.7 (16.0–57.4)	1.71 (1.46–2.09)	0.012*
**Physical contact with the case**					
Absent	643	196	23.8 (10.7–36.7)	Ref	
Present	2	1	40.6 (-28.5–97.1)	2.28 (1.14–36.64)	0.048*
**Wearing a mask when coming in contact with case**					
No	494	155	40.2 (6.9–73.7)	Ref	
Yes	144	41	37.1 (3.5–70.5)	0.87 (0.57–0.97)	0.015*
Sometimes	7	1	19.8 (-20.4–60.0)	0.36 (0.04–3.05)	0.308

*Significant risk factors associated with SIR (p<0.05)

^#^OR (95% CI)–Odds Ratio (95% Confidence Interval)

The significant factors (p<0.05) in univariate analysis were run through binomial regression model to identify the independent risk factors associated with secondary infection within households. After adjusting for other risk factors, the adjusted OR were as follows: household contacts who were the primary care giver of the case (OR = 1.60 [95% CI: 1.42–2.85]), who were with underlying medical conditions (OR = 1.83 [95% CI: 1.09–3.05]), overcrowded households (OR = 1.43 [95% CI: 1.13–2.26]), whose index cases were mildly symptomatic (OR = 1.44 [95% CI: 1.09–2.09]) and moderately/severely symptomatic (OR = 1.48 [95% CI: 1.26–1.89]), who were sharing toilet with the index cases (OR = 1.82 [95% CI: 1.29–2.93]) remained significantly associated with increased risk for COVID 19 as independent risk factors. Also who were wearing a mask when coming in contact with the case (OR = 0.86 [95% CI: 0.56–0.93]) was significantly associated with decreased risk for COVID 19 (Table 3).

**Table 3:** Risk factors associated with secondary infection rate of COVID-19 in households of CRHSP, Ballabgarh, Haryana, 2021.

Variables	aOR	95% CI	p value
**Relationship to index case**			
Others	Ref		
Primary care giver	1.60	1.42–2.85	0.004*
**Overcrowding**			
No	Ref		
Yes	1.43	1.13–2.26	0.012*
**Clinical severity of index case**			
Asymptomatic	Ref		
Mild	1.44	1.01–2.09	0.042*
Moderate/Severe	1.48	1.26–1.89	0.021*
**Underlying medical conditions**			
Absent	Ref		
Present	1.83	1.09–3.05	0.020*
**Sharing a toilet**			
Absent	Ref		
Present	1.82	1.29–2.93	0.040*
**Physical contact with the case**			
Absent	Ref		
Present	1.57	0.09–36.45	0.752
**Wearing mask when coming in contact with case**			
No	Ref		
Yes	0.86	0.56–0.93	0.036*
Sometimes	0.32	0.03–2.81	0.304

*Significant risk factors associated with COVID 19 (p<0.05)

^#^aOR (95% CI)–Adjusted Odds Ratio (95% Confidence Interval)

### Discussion

Households offer an ideal setting for assessing the infectivity and transmissibility of SARS-CoV-2 and its associated risk factors [1]. To assess this, we conducted a prospective case- ascertained study of identified household contacts of a RT- PCR confirmed SARS-CoV-2 infection in Ballabgarh, Haryana. The secondary infection rate (SIR) was 30.5% and secondary clinical attack rate was 9.3%. The risk factors associated with secondary transmission of COVID-19 were the household contacts who were the primary care giver of the case, who were with underlying medical conditions, overcrowded households, whose index cases were symptomatic, who were sharing a toilet with the index cases and also who were not wearing mask when coming in contact with the case. The median incubation period was 4 days (4, 5). The mean serial interval was 6.4 days and the median (IQR) serial interval was 5 days (5,7).

In our study, assuming that all the secondary cases were infected by their index cases, the secondary infection rate was 30.5% (95% CI: 27.1–34.1%). The SIR in our study corroborates with the studies done in Wuhan (30%) [19], Zhuhai (32.4%) [9] and Zhejiang (34.43%) [21] provinces of China and North Carolina (32%) [18] and Utah and Wisconsin province (29%) [20]. However our estimate was lower when compared with that of Tennessee and Wisconsin of USA (53%) [17]. Majority of the studies reported lower estimates of secondary infection in the range of 4.6%– 23.0% in various provinces of China and USA [10, 16]. The pooled estimates of SIR from meta-analysis studies also were also lower as 16.6% - 18.1% [1, 22, 23] with significant heterogeneity across the studies ranging from 3.9% to 54.9% [23].

The difference in secondary infection rates between our study and others published may have been due to various reasons including approaches of identifying or defining secondary cases, features of the cases investigated, environmental and behavioural differences, control measures of that geographical area for mitigating the disease spread and more importantly the study design [9, 22]. The published studies were a combination of both retrospective and prospective study designs, with the former comprising most of them. As expected, studies with increased frequency of testing regardless of symptom status and longer follow up of the contacts would generally report higher infection rates [18]. Several studies have reported lower estimates of household transmission, mainly from contact tracing activities, with limited follow-up and testing of household contacts or delayed enrolment relative to index patient identification [17]. Our study was a prospective one with longer follow-up (28 days), including weekly RT- PCR testing up to 14 days, combined with antibody testing at day 28. Therefore, the chance of higher secondary infection rates relative to other studies is probable. Studies conducted similarly also reported higher SIR as in North Carolina (32%) [18], Utah and Wisconsin provinces (29%) [20], and Tenesse and Wisconsin of USA (53%) [17] which were prospective and also included serological testing for antibody detection. However, the possibility of secondary cases identified later during follow-up acquired from the community other than the index cases cannot be ruled out. The actual SIR may be likely lower than that observed in our study. Nonetheless, the still high household SIR observed in our study underscores the need for effective control measures for SARS-CoV-2 within households.

In this study, the secondary clinical attack rate was 9.3% (95% CI: 7.2–11.8%). This study’s secondary clinical attack rate is much higher when compared to a study done in Kerala, India (2.6%) and in Singapore (5.9%) [7, 24]. This higher rate may be attributed because the studies were cross-sectional or retrospective.

In this study, we estimated that the median (IQR) incubation period of COVID-19 was 4 days (4,5). This is in line with the estimate reported in 30 provinces of China (4 days) [26] and Zhuhai, China (4.3 days) [9] and slightly lower than those reported outside of Wuhan (5.1 days) [27] and in Wuhan (5.2 days) [28]. Meta-analysis across various regions revealed an estimate between 4.0 to 9.0 days [29, 30]. Similarly, the median (IQR) serial interval calculated in our study was 5 days (5,7) which is in line with the estimate reported in Zhuhai, China (5.1 days) [9] and slightly higher than those reported in mainland China (4.6 days) [25]. Serial interval across various regions of the world revealed an estimate between 4.0 to 8.0 days [25, 29, 30]. The incubation period reported by WHO estimates an average of 5–6 days up to 14 days [31]. A serial interval shorter than the incubation period implies the pre-symptomatic transmission during the incubation period. Therefore it is difficult to contain the epidemic, as an infectious person during this period is hard to identify and is likely to be moving around [25, 29].

In this study, the SIR was found to be higher in the elderly above 60 years of age (42.1% [31.0–53.2%]) though it did not reach a statistical significance. And SIR in children less than 10 years (31.7% [20.3–43.2%]) is higher than those of adolescents (28.3% (20.5–36.2%]) and young and middle aged adults (28.8% [24.2–33.3%]) and it is in line with the results of other studies conducted across the globe [1, 5, 6, 10, 20, 22, 23]. A study in South Korea noted relatively high transmission from index cases for those aged 10 to 19 years. Although children seem to be at reduced risk for symptomatic disease, it is still unclear whether they shed virus similarly to adults [1, 23, 32].

In this study, the primary care giver of the index case showed a higher SIR (36.8% [31.0–42.5%]) compared to others (26.1% [21.6–30.5%]). The former had 1.6 times more risk of becoming the secondary case (OR 1.60, 95% CI: 1.42–2.85, p<0.05). The primary care giver was majorly the spouse who looked after the COVID positive case. This corroborates with the previously reported studies [1, 6, 10, 20]. Spouses were at higher risk than other household contacts possibly due to their active involvement in the care taking activities of the positive cases. This may have resulted in prolonged, very close physical contact with the index cases and hence longer or more direct exposure to the index case. Another household factor that was a significant risk factor in our study was overcrowding at the households. Secondary cases residing in overcrowded households showed a higher SIR (SIR 34.8% [95% CI: 26.0–43.6%]) compared to non-overcrowded ones (SIR 29.6% [95% CI: 25.8–33.5%]) and the former had 1.43 times more risk of becoming the secondary case (OR 1.43, 95% CI: 1.13–2.26, p<0.05). This is supported by the study in Israel which showed that relatively large numbers of cohabitants living in a close-knit community contributed to the study’s reported high household SIR [22]. In contrary to this, a study in Kerala reported a mild negative correlation between the number of positive COVID-19 cases and rooms per person denoting that even though overcrowding should have increased the number of cases there can be multiple other associated concealed or unidentified factors and natural immunity or resistance to the virus which might be playing a role in determining the rate of household transmission of COVID–19 [5].

Another significant risk factor was that household contacts with underlying medical conditions had higher SIR (SIR 42.1% (95% CI: 31.0–53.2%]) compared with those who did not (SIR 28.9% [95% CI: 25.3–32.7%]) and the former had 1.83 times more risk of becoming the secondary case (OR 1.83, 95% CI: 1.09–3.05, p<0.05). This is in line with most of the studies that stated old age and comorbid conditions are independent risk factors of COVID-19 infections explaining the higher transmission rate in elderly in household SAR as well [6, 9, 20].

One interesting risk factor that was found to be significantly associated with secondary disease transmission in COVID was sharing the same toilet as used by the positive case of COVID. Sharing a toilet with index case showed a higher SIR (36.7% [95% CI: 16.0–57.4%]) and such contacts had 1.82 times more risk of contracting the disease (OR = 1.82, 95% CI: 1.29–2.93, p<0.05). The possible reasons may be the lots of contact surfaces (handles, taps, etc) where the virus may survive and pass from one person to another; the presence of cold and wet surfaces which are conducive to viral survival; flushing toilets that can contribute to aerosol transfer of the virus to the air and contamination of adjacent surfaces; the possibility of non-disinfected toilets [33].

In this study, household contacts who were wearing mask when coming in contact with the index case showed a lower SIR (37.1% [3.5–70.5%]) than those who were not wearing mask when coming in contact with the primary case. Household contacts who were wearing masks had decreased risk for COVID 19 (OR = 0.86, 95% CI: 0.56–0.93, p<0.05). This in fact is supported by various other studies [9, 10, 20, 22, 34]. A study conducted in Beijing, China reported face masks were 79% effective in preventing transmission [34]. The findings inform universal face mask use, not just in public spaces, but inside the household with members at risk of infection reducing the risk of transmission. Studies also showed that non-pharmacological interventions like separate dining, indoor isolation, ventilation and disinfection, index patient living alone, and social distancing are effective at preventing transmission, even in homes that are crowded and small houses [9, 10, 34].

This study has few strengths as this is one among the first few studies conducted prospectively in investigating the household transmission investigation of COVID 19 in India with adequate follow ups up to 28 days and adequate testing with RT PCR and serology. But it is not free of limitations. We assumed that only household transmission was responsible for secondary infections among household contacts. So the household SIR could be an overestimation, although simultaneous quarantine orders for household contacts could have limited the community exposures. Also, the initial household member who experienced symptoms and tested positive was considered the index patient. Still, it is possible that other household members were infected concurrently or even prior to the index case but developed symptoms at different times or remained asymptomatic thus not diagnosed. Also, in estimation of incubation period, we have not used any statistical models and considered median incubation period. Since this is a follow up study, there was an issue with loss to follow up and few sample refusals which could have underestimated our estimation of the SIR.

## Conclusion and recommendations

Our study highlights the important aspects of secondary infection rates, risk factors, and epidemiological parameters associated with COVID-19 in household contacts with recent exposure to patients with SARS-COV-2. Despite household contacts developing COVID infection, a significant number of individuals did not develop an infection in spite of sharing the same household, which indicates a role of individual-specific natural immunity for resistance to COVID-19. As transmission occurs favourably within households, immediate isolation of the index case with separate dining, sleep room, lavatory, adequate ventilation and wearing masks is mandatory. Along with isolation of index cases, following infection control activities (handwashing, wearing masks, social distancing) customarily by the household contacts especially by the spouse and those with any comorbid conditions. The household contacts should be receptive to following all the COVID prevention protocols before the local health authorities intervene in decreasing the disease transmission among them.

## References

[1] Madewell ZJ, Yang Y, Longini IM, Halloran ME, Dean NE. Household Transmission of SARS-CoV-2: A Systematic Review and Meta-analysis. JAMA network open. 2020 Dec 1;3(12):e2031756. doi: 10.1001/jamanetworkopen.2020.31756 33315116 PMC7737089

[2] Coronavirus (COVID-19) events as they happen. Available from: https://www.who.int/emergencies/diseases/novel-coronavirus-2019/events-as-they-happen. Accessed on 18th September, 2022.

[3] WHO Coronavirus Disease (COVID-19) Dashboard. Available from: https://covid19.who.int. Accessed on 7^th^ March, 2023.

[4] World Health Organization. Household transmission investigation protocol for coronavirus disease 2019 (COVID-19), 23 March 2020. World Health Organization; 2020.

[5] Dutta S, Kaur RJ, Bhardwaj P, Charan J, Bist SK, Detha MD, Kanchan T, et al. Household Transmission of COVID-19: A Cross-Sectional Study. Infection and drug resistance. 2020;13:4637–42. doi: 10.2147/IDR.S285446 33380813 PMC7767703

[6] Shah K, Saxena D, Mavalankar D. Secondary attack rate of COVID-19 in household contacts: a systematic review. QJM-INT J MED. 2020 Dec;113(12):841–50. doi: 10.1093/qjmed/hcaa232 32726452 PMC7454929

[7] Saraswathy AS, Sanya R, Kumaran JA. Secondary attack rate of COVID-19; analysis of contacts of COVID-19 cases admitted in a tertiary care centre, Northern District of Kerala: a cross-sectional study. Int J Community Med Public Health 2020;7:5111–4.

[8] Jing L, Liu M, Zhang Z, Fang L, Yuan J, Zhang A et al. Household secondary attack rate of COVID-19 and associated determinants in Guangzhou, China: a retrospective cohort study. Lancet Infect Dis Jun 2020. doi: 10.1016/S1473-3099(20)30471-0 32562601 PMC7529929

[9] Wu J, Huang Y, Tu C, Bi C, Chen Z, Luo L et al. Household Transmission of SARS-CoV-2, Zhuhai, China, 2020. Clinical Infectious Diseases. 2020;71(16):2099–2108. doi: 10.1093/cid/ciaa557 32392331 PMC7239243

[10] Li W, Zhang B, Lu J, Liu S, Chang Z, Peng C, et al. Characteristics of household transmission of COVID-19. Clinical Infectious Diseases. 2020 Oct 15;71(8):1943–6. doi: 10.1093/cid/ciaa450 32301964 PMC7184465

[11] Rosenberg ES, Dufort EM, Blog DS, et al. New York State Coronavirus 2019 Response Team. COVID-19 testing, epidemic features, hospital outcomes, and household prevalence, New York State-March 2020. *Clin Infect Dis*. 2020:ciaa549. doi: 10.1093/cid/ciaa549 PMC7239264 32382743

[12] Laxminarayan R, Wahl B, Dudala SR, Gopal K, Neelima S, Reddy KJ, Radhakrishnan J, Lewnard JA. Epidemiology and transmission dynamics of COVID-19 in two Indian states. Science. 2020 Nov 6;370(6517):691–7. doi: 10.1126/science.abd7672 33154136 PMC7857399

[13] Ministry of Health and Family welfare, Government of India. Clinical management protocol: COVID-19, 13 June 2020. Government of India; 2020.

[14] Park K. Environment and health. In: Park’s Textbook of Preventive and Social Medicine, 25th ed. Jabalpur, India: M/S Banarasidas Bhanot Publishers; 2019.

[15] Wantai SARS-CoV-2 Manual Diagnostic Kits. WANTAI SARS-CoV-2 Ab ELISA. Beijing Wantai Biological Pharmacy Enterprise Co., Ltd. 2020. https://www.ystwt.cn/wp-content/uploads/2020/05/Brochure-Wantai-SARS-CoV-2-Ab-ELISA.pdf. Accessed on 10^th^ June, 2021.

[16] Li F, Li YY, Liu MJ, Fang LQ, Dean NE, Wong GW, et al. Household transmission of SARS-CoV-2 and risk factors for susceptibility and infectivity in Wuhan: a retrospective observational study. The Lancet Infectious Diseases. 2021 May 1;21(5):617–28. doi: 10.1016/S1473-3099(20)30981-6 33476567 PMC7833912

[17] Grijalva CG, Rolfes MA, Zhu Y, McLean HQ, Hanson KE, Belongia EA, et al. Transmission of SARS-COV-2 infections in households—Tennessee and Wisconsin, April–September 2020. Morbidity and Mortality Weekly Report. 2020 Nov 6;69(44):1631–34. doi: 10.15585/mmwr.mm6944e1 33151916 PMC7643897

[18] Cerami C, Popkin-Hall ZR, Rapp T, Tompkins K, Zhang H, Muller MS, et al. Household transmission of SARS-CoV-2 in the United States: living density, viral load, and disproportionate impact on communities of color. Clinical infectious diseases: an official publication of the Infectious Diseases Society of America. 2021 Aug 12:ciab701. 10.1093/cid/ciab701 PMC8436395 34383889

[19] Wang Z, Ma W, Zheng X, Wu G, Zhang R. Household transmission of SARS-CoV-2. Journal of Infection. 2020 Jul 1;81(1):179–82. doi: 10.1016/j.jinf.2020.03.040 32283139 PMC7151261

[20] Lewis NM, Chu VT, Ye D, Conners EE, Gharpure R, Laws RL, et al. Household transmission of severe acute respiratory syndrome coronavirus-2 in the United States. Clinical Infectious Diseases. 2021 Oct 1;73(7):e1805–13. doi: 10.1093/cid/ciaa1166 33185244 PMC7454394

[21] Sun WW, Ling F, Pan JR, Cai J, Miao ZP, Liu SL, Cheng W, Chen EF. Epidemiological characteristics of COVID-19 family clustering in Zhejiang Province. Zhonghua yu Fang yi xue za zhi [Chinese Journal of Preventive Medicine]. 2020 Jun 1;54(6):625–9. doi: 10.3760/cma.j.cn112150-20200227-00199 32171192

[22] Fung HF, Martinez L, Alarid-Escudero F, Salomon Ja, Studdert DM, Andrews JR, et al. The household secondary attack rate of SARS-CoV-2: a rapid review. Clin Infect Dis. https://doi.org/10. 2020;1093. 10.1093/cid/ciaa1558 PMC7665336 33045075

[23] Koh WC, Naing L, Chaw L, Rosledzana MA, Alikhan MF, et al. What do we know about SARS-CoV-2 transmission? A systematic review and meta-analysis of the secondary attack rate and associated risk factors. PloS one. 2020 Oct 8;15(10):e0240205. doi: 10.1371/journal.pone.0240205 33031427 PMC7544065

[24] Ng OT, Marimuthu K, Koh V, Pang J, Linn KZ, Sun J, et al. SARS-CoV-2 seroprevalence and transmission risk factors among high-risk close contacts: a retrospective cohort study. The Lancet infectious diseases. 2021 Mar 1;21(3):333–43. doi: 10.1016/S1473-3099(20)30833-1 33152271 PMC7831879

[25] Guan WJ, Ni ZY, Hu Y, Liang WH, Ou CQ, He JX, et al. Clinical characteristics of coronavirus disease 2019in China. N Engl J Med 2020; 382:1708–20. doi: 10.1056/NEJMoa2002032 32109013 PMC7092819

[26] Lauer SA, Grantz KH, Bi Q, Jones FK, Zheng Q, Meredith HR et al. The incubation period of coronavirus disease 2019 (COVID-19) from publicly reported confirmed cases: estimation and application. Ann Intern Med 2020 May 5;172(9):577–82. doi: 10.7326/M20-0504 32150748 PMC7081172

[27] Li Q, Guan X, Wu P, Wang X, Zhou L, Tong Y, et al. Early transmission dynamics in Wuhan, China, of novel coronavirus-infected pneumonia. N Engl J Med 2020; 382:1199–207. doi: 10.1056/NEJMoa2001316 31995857 PMC7121484

[28] Nishiura H, Linton NM, Akhmetzhanov AR. Serial interval of novel coronavirus (COVID-19) infections. Int J Infect Dis 2020; 93:284–6. doi: 10.1016/j.ijid.2020.02.060 32145466 PMC7128842

[29] Park M, Cook AR, Lim JT, Sun Y, Dickens BL. A systematic review of COVID-19 epidemiology based on current evidence. J. Clin.med. 2020 Apr;9(4):967. doi: 10.3390/jcm9040967 32244365 PMC7231098

[30] Alene M, Yismaw L, Assemie MA, Ketema DB, Gietaneh W, Birhan TY. Serial interval and incubation period of COVID-19: a systematic review and meta-analysis. BMC Infectious Diseases. 2021 Dec;21(1):1–9. 33706702 10.1186/s12879-021-05950-x PMC7948654

[31] World Health Organization. Transmission of SARS-CoV-2: implications for infection prevention precautions: scientific brief, 09 July 2020. World Health Organization, Geneva; 2020.

[32] Zhu Y, Bloxham CJ, Hulme KD, Sinclair JE, Tong ZW, Steele LE, et al. Children are unlikely to have been the primary source of household SARS-CoV-2 infections. The Lancet infectious diseases. 2020. Available at SSRN: https://ssrn.com/abstract=3564428 or 10.2139/ssrn.3564428.

[33] Technical advisory group. SARS-CoV-2 transmission risk in Public toilets. 12 February 2021. Available at https://gov.wales/sites/default/files/publications/2021-03/technical-advisory-group-sars-cov-2-transmission-risk-in-public-toilets.pdf.

[34] Wang Y, Tian H, Zhang L, Zhang M, Guo D, Wu W, et al. Reduction of secondary transmission of SARS-CoV-2 in households by face mask use, disinfection and social distancing: a cohort study in Beijing, China. BMJ global health. 2020 May 1;5(5):e002794. 10.1136/bmjgh-2020-002794 PMC7264640 32467353

